# Complete genome sequences of *Staphylococcus epidermidis* phages Cicami, Sazerac, Southeast, Slasher, Spartan, and Undine

**DOI:** 10.1128/mra.01000-25

**Published:** 2026-03-18

**Authors:** Courtney M. Hill, Lance J. Pollitz, Tori A. Boyle, Ashley Akrami, Connor Briley, Shannon Cichanski, Anthony DeMarco, Kevin Gonzalez, Norma Rojas Nava, Yusuf Qavi, Karla Ybanez, Barbaros Aslan, Asma Hatoum-Aslan

**Affiliations:** 1 Department of Microbiology, University of Illinois at Urbana-Champaign 14589, Urbana, Illinois, USA; 2 School of Molecular and Cellular Biology, University of Illinois at Urbana-Champaign 14589, Urbana, Illinois, USA; Portland State University, Portland, Oregon, USA

**Keywords:** bacteriophages, bacteriophage therapy, coagulase-negative staphylococci

## Abstract

Staphylococci are opportunistic pathogens that cause a variety of antibiotic-resistant infections, and staphylococcal viruses (phages) can be harnessed as alternative therapeutics. Here, we report genome sequences of six *Staphylococcus epidermidis* phages with siphovirus morphology that lack proteins associated with virulence and lysogeny. Our observations suggest potential uses in therapeutic applications.

## ANNOUNCEMENT

*Staphylococcus epidermidis* is a commensal opportunistic pathogen commonly associated with infections on indwelling medical devices (1), and phage therapy has gained increasing interest as a strategy to combat implant-associated infections (2–4). While known staphylococcal phages with myovirus and podovirus morphologies are strictly lytic and constitute ideal therapeutic candidates, the majority of sequenced staphylococcal phages with siphovirus morphology are lysogenic and considered unsuitable for therapeutic applications. However, rare members of this group are devoid of recognizable integrases and thought to be strictly lytic (5), making such phages worthy of investigation as therapeutic candidates.

**Fig 1:**
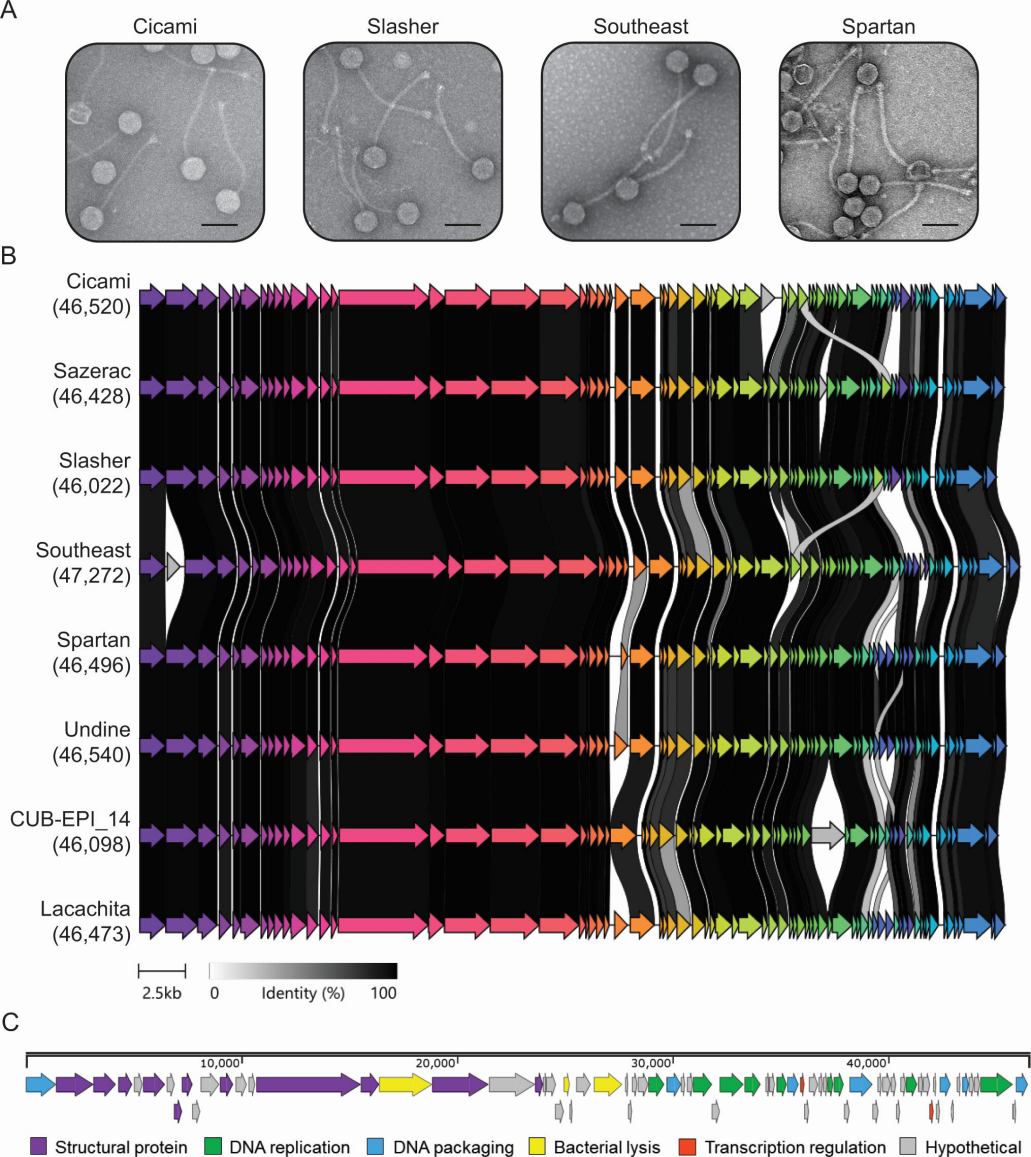
Phenotypic and genotypic features of *S. epidermidis* phages Cicami, Sazerac, Slasher, Southeast, Spartan, and Undine. (**A**) Transmission electron micrographs of representative phages. High-titer phage lysates were deposited on formvar-coated TEM grids, stained with 2% uranyl acetate, and imaged with accelerating voltages of 60 (Cicami and Southeast) and 120 kV (Slasher and Spartan). Scale bar = 100 nm. (**B**) Gene cluster alignment of discovered phages with related phages CUB-EPI_14 and Lacachita. Numbers in parentheses indicate genome lengths in nucleotides, and open reading frames (ORFs) are shown as arrows. Homologous proteins are similarly colored with gray connectors indicating percent protein identity according to the legend at the bottom. The alignment was generated using Clinker (13). (**C**) Genome map of phage Cicami showing predicted protein functions.

Here, we report the complete genome sequences of six *S*. *epidermidis* phages with siphovirus morphology (Fig. 1 and Table 1). The phages were isolated from untreated wastewater collected from wastewater treatment facilities in Tuscaloosa, Alabama (Cicami and Southeast) and Urbana-Champaign, Illinois (Sazerac, Slasher, Spartan, and Undine) (Table 1). All phages were isolated using as ‘bait’ an immunocompromised host, *S. epidermidis* LM1680, a variant of *S. epidermidis* RP62a (6) that has lost the majority of its anti-phage defenses (7, 8). Phages were enriched by culturing wastewater (pre-treated with 2% chloroform) with the host at 37°C in tryptic soy broth (Difco) supplemented with 5 mM CaCl_2_ for three consecutive nights as described in reference 9. Traditional assays using the double-agar overlay method were used to visualize and purify phage plaques and amplify phages to high titer (>10^9^ particles per milliliter) (9). Phages were imaged using transmission electron microscopy (TEM), and genomic DNA was extracted using the Wizard DNA Extraction Kit (Promega) according to the manufacturer’s instructions. Cicami, Sazerac, Southeast, Spartan, and Undine sample libraries were prepared using an Illumina DNA Prep Kit and sequenced on an Illumina NextSeq 2000. Slasher’s sample library was prepared using the tagmentation- and PCR-based Illumina Prep Kit and sequenced on an Illumina NovaSeq X Plus. Demultiplexing, quality control, and adapter trimming were performed with bcl-convert (v3.9.3) and bcl-convert1 (v4.2.4). Sequencing reads were assembled using SPAdes v3.15.3 (10), and assembly graphs were visualized in Bandage v0.8.1 (11) using default parameters. All phages were assembled into single circular contigs (Table 1), and junctions between contig ends were confirmed by PCR and Sanger sequencing. Genes and protein functions were predicted using Phynteny and Phold as part of the rapid phage annotation tool, Pharokka (12).

**TABLE 1:** Additional information pertaining to sample collection, Illumina sequencing, and genetic features of the six *S*. *epidermidis* phages reported in this study

Phage	Collection date	Collection site	GPS coord.	GC content (%)	ORFs	ORFs with known functions	Depth of coverage	Raw reads	Raw read files
Cicami	January 2020	Hilliard N Fletcher Water Resource Facility	33.17045, −87.56117	34.3	73	32	2,499.10	1,624,291	SRR34308873
Sazerac	October 2021	Urbana Champaign Sanitary District SW Plant	40.08465, −88.33282	36.0	71	23	3,640.30	1,828,003	SRR19241993
Slasher	January 2025	Urbana Champaign Sanitary District NE Plant	40.12062, −88.19589	36.3	72	28	2,599.97	2,972,425	SRR34308870
Southeast	January 2018	Hilliard N Fletcher Water Resource Facility	33.17045, −87.56117	35.0	70	24	2,444.00	1,370,722	SRR23857886
Spartan	January 2022	Urbana Champaign Sanitary District NE Plant	40.12062, −88.19589	35.5	73	30	3,841.00	1,930,419	SRR34308872
Undine	January 2022	Urbana Champaign Sanitary District NE Plant	40.12062, −88.19589	35.2	73	31	3,671.23	1,828,361	SRR34308871

All six phages have double-stranded DNA genomes between 46,000 and 48,000 nucleotides in length and encode 70–80 genes (Table 1 and Fig. 1B). Importantly, the phages lack identifiable genes associated with virulence and lysogeny as evidenced by the absence of such predicted domains following genome annotation. Their nucleotide sequences share >96% nucleotide identity when compared to each other and >90% nucleotide identity when compared with two previously reported *S. epidermidis* phages—CUB-EPI_14 (ON325435.2) and Lacachita (OP142323) (14, 15). While CUB-EPI_14 is thought to be suitable for therapeutics (14), Lacachita is capable of transducing antibiotic resistance genes, making it a poor therapeutic candidate (15). Thus, further studies are required on the lifestyle and transducing potential of Cicami, Sazerac, Slasher, Southeast, Spartan, and Undine to accurately assess their therapeutic utility.

## Data Availability

The sequencing reads for Cicami, Slasher, Southeast, Spartan, and Undine were deposited in GenBank under the BioProject PRJNA943272. The sequencing reads for Sazerac were deposited in GenBank under the BioProject PRJNA837416. The complete genome sequences of the phages can be found under accession numbers PV956268, PV956269, OQ623150, PV956270, PV956271, and ON550478. The versions described in this paper are the first versions.
